# Clinical significance of elevated D-dimer in emergency department patients: a retrospective single-center analysis

**DOI:** 10.1186/s12245-024-00620-6

**Published:** 2024-04-03

**Authors:** Mohammed Alshalhoub, Faisal Alhusain, Feras Alsulaiman, Abdulaziz Alturki, Saud Aldayel, Majid Alsalamah

**Affiliations:** 1 Emergency Medicine Department, Ministry of the National Guard - Health Affairs, Riyadh, Saudi Arabia; 2 King Abdullah International Medical Research Center, Riyadh, Saudi Arabia; 3 College of Medicine, King Saud bin Abdulaziz University for Health Sciences, Riyadh, Saudi Arabia

## Abstract

**Introduction:**

D-dimer is a marker of coagulation and fibrinolysis widely used in clinical practice for assessing thrombotic activity. While it is commonly ordered in the Emergency Department (ED) for suspected venous thromboembolism (VTE), elevated D-dimer levels can occur due to various other disorders. The aim of this study was to find out the causes of elevated D-dimer in patients presenting to a large ED in Saudi Arabia and evaluate the accuracy of D-dimer in diagnosing these conditions.

**Methods:**

Data was collected from an electronic hospital information system of patients who visited the ED from January 2016 to December 2022. Demographic information, comorbidities, D-dimer levels, and diagnoses were analyzed. Statistical analysis was performed using the SPSS software. The different diagnoses associated with D-dimer levels were analyzed by plotting the median D-dimer levels for each diagnosis category and their interquartile ranges (IQR). The receiver operating characteristic (ROC) curves were calculated and their area under the curve (AUC) values were demonstrated. The optimal cut-off points for specific diseases were determined based on the ROC analysis, along with their corresponding sensitivities and specificities.

**Results:**

A total of 19,258 patients with D-dimer results were included in the study. The mean age of the participants was 50 years with a standard deviation of ± 18. Of the patients, 66% were female and 21.2% were aged 65 or above. Additionally, 21% had diabetes mellitus, 20.4% were hypertensive, and 15.1% had been diagnosed with dyslipidemia. The median D-dimer levels varied across different diagnoses, with the highest level observed in aortic aneurysm 5.46 g/L. Pulmonary embolism (PE) and deep vein thrombosis (DVT) were found in 729 patients (3.8%) of our study population and their median D-dimer levels 3.07 g/L (IQR: 1.35–7.05 g/L) and 3.36 g/L (IQR: 1.06–8.38 g/L) respectively. On the other hand, 1767 patients (9.2%) were diagnosed with respiratory infections and 936 patients (4.9%) were diagnosed with shortness of breath (not specified) with median D-dimer levels of 0.76 g/L (IQR: 0.40–1.47 g/L) and 0.51 g/L (IQR: 0.29–1.06 g/L), respectively. D-dimer levels showed superior or excellent discrimination for PE (AUC = 0.844), leukemia (AUC = 0.848), and aortic aneurysm (AUC = 0.963). DVT and aortic dissection demonstrated acceptable discrimination, with AUC values of 0.795 and 0.737, respectively. D-dimer levels in respiratory infections and shortness of breath (not specified) exhibited poor to discriminatory performance.

**Conclusion:**

This is the first paper to identify multiple causes of elevated D-dimer levels in Saudi Arabia population within the ED and it clearly highlights their accurate and diagnostic values. These findings draw attention to the importance of considering the specific clinical context and utilizing additional diagnostic tools when evaluating patients with elevated D-dimer levels.

## Introduction

D-dimer is a marker of coagulation and fibrinolysis, providing a rapid assessment of thrombotic activity. It has multiple uses in clinical practice and has been adopted by Wells’ criteria for deep vein thrombosis (DVT) and pulmonary embolism (PE) [1]. In the Emergency Department (ED), it is commonly ordered when there is a suspicion of venous thromboembolism (VTE). However, it can be high due to other disorders like infections, VTE, heart failure, trauma, and diseases like coronavirus disease of 2019 (COVID-19) [2, 3]. It can also be an indicator of recurrent VTE, as was studied by Halaby et al. [4] A meta-analysis published in 2022 found that D-dimer has prognostic value in pneumonia [5]. In addition, it has been tested as a marker of recurrent myocardial infarction [6]. Specifically, COVID-19 infection is often associated with elevated D-dimer levels due to the virus’s ability to trigger a hypercoagulable state, increasing the risk of blood clots. This makes D-dimer testing a valuable tool in identifying and managing potential complications in COVID-19 patients. Moreover, D-dimer can be useful in ruling out life threatening diagnoses such as VTE, especially in patients with low risk for VTE, as recommended by multiple international guidelines [7]. However, multiple studies have shown that D-dimer levels increase with age [8, 9]. Therefore, D-dimer levels should be interpreted with caution in older adults. The use of D-dimer levels can go beyond VTEs, as it can be helpful in ruling out aortic dissection in low-risk patients, as proven by recent studies [10]. However, the diagnostic value and the prognostic value of D-dimer can differ in different types of aortic dissection [11]. Due to the numerous diseases that increase D-dimer levels, they can be interpreted as false-positive results, which can result in unnecessary testing and treatment. Proper identification of all of these conditions that increase D-dimer would lead to better clinical practice. Therefore, the aim of this study was to find out the causes of elevated D-dimer in patients presenting to a large ED in Saudi Arabia and evaluate the accuracy of D-dimer in these conditions.

## Methods

This was a single-center retrospective cohort study based on data extracted from an electronic hospital information system (BESTCare) of patients presenting to the ED of King Abdulaziz Medical City-Riyadh (KAMC-R) in Saudi Arabia. The hospital has a bed capacity of 1501 with more than 100 emergency beds. The inclusion criteria included Saudi and non-Saudi patients who were over 18 years old and had a D-dimer test ordered during their ED visit. Pregnant patients were excluded from the study as the physiological changes in pregnancy affects the D-dimer level. The following data were collected: age, gender, comorbidities, D-dimer level, and diagnosis. D-dimer analysis in our institution is performed via a laboratory-based INNOVANCE® D-Dimer assay, not through a point-of-care test. While specific instrumentation may vary across different laboratories, the employed assay adheres to our established cut-off value of 0.5 µg/mL. Consequently, values below this threshold are considered within the reference range, whereas elevated levels (> 0.5 µg/mL) warrant further evaluation in the context of the patient’s clinical presentation and additional relevant diagnostic tests.

Data were analyzed with the Statistical Package for the Social Sciences (IBM Corp. Released 2013. IBM SPSS Statistics for Windows, Version 22.0. Armonk, NY). The demographic information and baseline characteristics were summarized and are reported in frequency, and the numerical variables as mean and standard deviation (SD) and interquartile ranges (IQR). The different diagnoses associated with D-dimer levels were analyzed by plotting the median D-dimer levels for each diagnosis category and their interquartile ranges were calculated. The receiver operating characteristic (ROC) curves were calculated by logistic regression to assess the discriminatory abilities of D-dimer levels for different diseases and their interpretations were according to Mandrekar et al. [12] The optimal cut-off points for specific diseases were determined based on the ROC analysis, along with their corresponding sensitivities and specificities.

The study protocol was approved by the institutional review board of KAMC-R. Patient confidentiality and privacy were strictly maintained during data collection and analysis. As this was a retrospective study using anonymized data, the need for informed consent was waived by the ethics committee.

## Results

Data was collected from January 1, 2016 to December 31, 2022. After excluding patients who did not meet the inclusion criteria, a total of 19,258 patients with D-dimer results were included in the study. The baseline characteristics and comorbidities of the patients is shown in Table 1. The mean age of the participants was 50 years with a standard deviation of ± 18. Of the patients, 66% were female and 21.2% were aged 65 or above. Additionally, 21% had diabetes mellitus, 20.4% were hypertensive, and 15.1% had been diagnosed with dyslipidemia.

**Fig 1:**
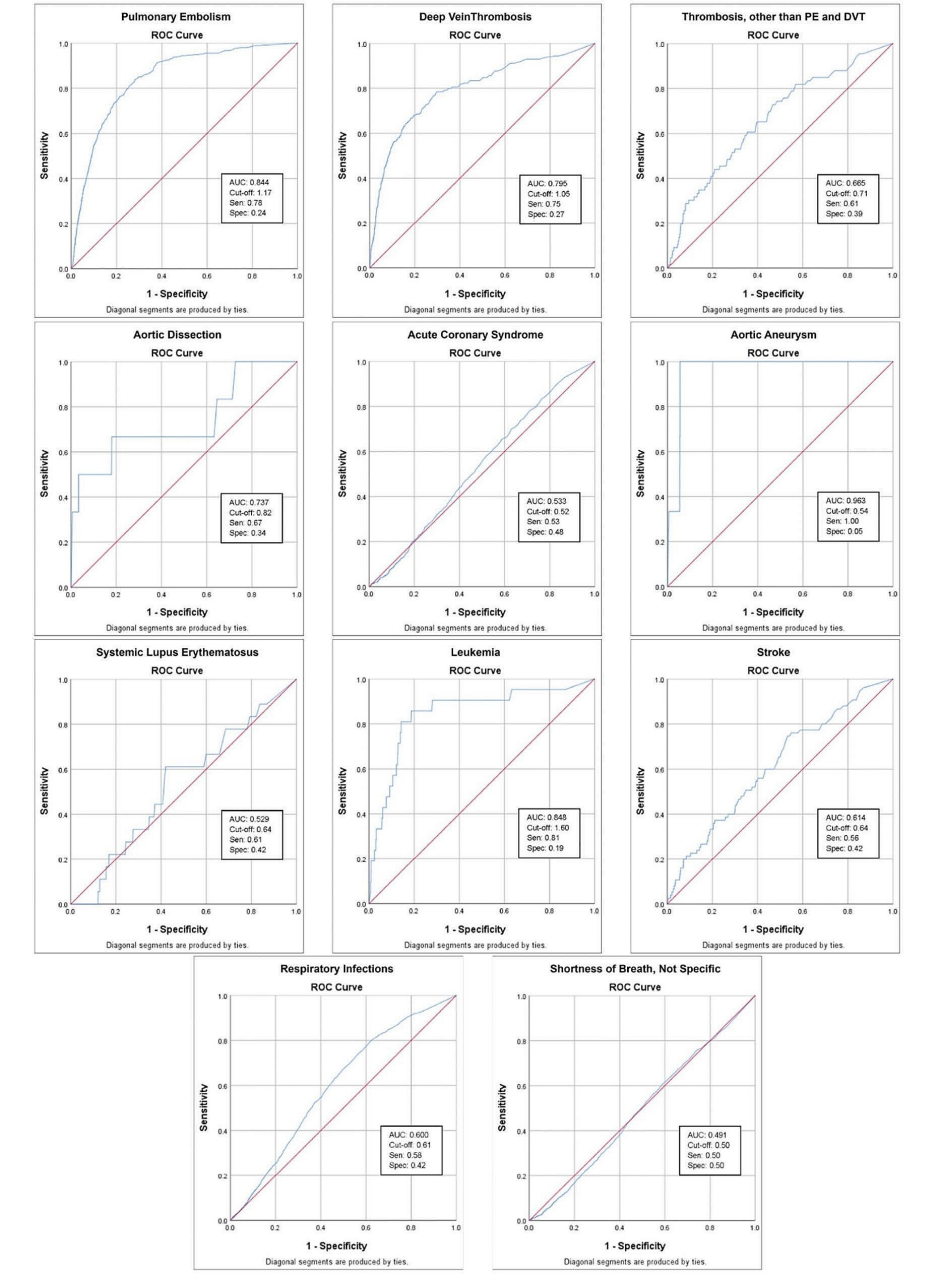
The discriminatory abilities of D-dimer levels for 11 different diseases using receiver operating characteristic (ROC) analysis and its optimal cut-off with their corresponding sensitivities (Sen) and specificities (Spec)

Table 2 identifies multiple diseases entities with elevated D-dimer. A total of 446 patients (2.3%) were diagnosed with PE and had a median D-dimer level of 3.07 g/L (IQR: 1.35–7.05 g/L). This was like the 283 patients (1.5%) who were DVT, with a median D-dimer level of 3.36 g/L (IQR: 1.06–8.38 g/L). Leukemia which was found in 21 patients (0.1%) gave similar results with a median D-dimer level of 3.33 g/L (IQR: 2.14–8.94 g/L). Aortic dissection was diagnosed in 66 patients (0.3%), with a median D-dimer level of 1.04 g/L (IQR: 0.49–4.05 g/L). A total of 552 patients (2.9%) were diagnosed with acute coronary syndrome (ACS) and had a median D-dimer level of 0.57 g/L (IQR: 0.32–1.21 g/L). This was similar to the 18 patients (0.1%) who were diagnosed with systemic lupus erythematosus (SLE), with a median D-dimer level of 0.67 g/L (IQR: 0.33–1.21 g/L). Stroke was found in 75 patients (0.4%) with a median D-dimer level of 0.82 g/L (IQR: 0.45–2.07 g/L). Finally, 1767 patients (9.2%) were diagnosed with respiratory infections and 936 patients (4.9%) were diagnosed with shortness of breath, not specified, with median D-dimer levels of 0.76 g/L (IQR: 0.40–1.47 g/L) and 0.51 g/L (IQR: 0.29–1.06 g/L), respectively.

**Table 1:** Baseline characteristics of the participants

Variable	N (%)
Age (mean ± SD)	50 ± 18
Gender, Female	12,719 (66)
Age more than 65	4076 (21.2)
Comorbidities:	
Asthma	2539 (13.2)
Atrial fibrillation	1043 (5.4)
Cancer	1670 (8.7)
Congestive heart failure	2379 (12.4)
Chronic obstructive pulmonary disease	443 (2.3)
Diabetes mellitus	4056 (21.1)
Dyslipidemia	2902 (15.1)
Hypertension	3920 (20.4)
Hyperthyroidism	166 (0.9)
Hypothyroidism	931 (4.8)
Ischemic heart disease	3145 (16.3)
Liver disease	25 (0.1)
Multiple sclerosis	53 (0.3)
Myocardial infarction	1020 (5.3)
Portal vein thrombosis	67 (0.3)
Pulmonary embolism	1437 (7.5)
Renal disease	1352 [7]
Stenosis	43 (0.2)
Transient ischemic attack	372 (1.9)
Unstable angina	499 (2.6)

**Table 2:** Different diagnoses and D-dimer levels

Variable		D-dimer				
		N	%	Median	25th Percentile	75th Percentile
Diagnosis	Others	15,085	78.3%	0.45	0.26	1.03
	Pulmonary embolism (PE)	446	2.3%	3.07	1.35	7.05
	Deep venous thrombosis (DVT)	283	1.5%	3.36	1.06	8.38
	Thrombosis, other than PE or DVT	6	0.0%	4.93	0.36	36.00
	Aortic Dissection	66	0.3%	1.04	0.49	4.05
	Acute coronary syndrome	552	2.9%	0.57	0.32	1.22
	Aortic aneurysm	3	0.0%	5.46	5.37	36.00
	Systemic lupus erythematosus	18	0.1%	0.67	0.33	1.21
	Leukemia	21	0.1%	3.33	2.14	8.94
	Stroke	75	0.4%	0.82	0.45	2.07
	Respiratory Infections	1767	9.2%	0.76	0.40	1.47
	Shortness of Breath, Not Specified	936	4.9%	0.51	0.29	1.06

The discriminatory abilities of D-dimer levels were assessed for 11 different diseases using ROC analysis. Figure 1 displays the ROC curves and the corresponding AUC values. D-dimer levels showed superior or excellent discrimination for PE (AUC = 0.844), leukemia (AUC = 0.848), and aortic aneurysm (AUC = 0.963). Optimal cut-off points for these diseases were determined, with corresponding sensitivities and specificities. DVT and aortic dissection demonstrated acceptable discrimination, with AUC values of 0.795 and 0.737, respectively. On the other hand, D-dimer levels in thrombosis other than PE or DVT, stroke, respiratory infections, SLE, and acute ACS exhibited poor discriminatory performance, while shortness of breath (not specified) showed no discrimination.

## Discussion

This is the first paper to identify multiple causes of elevated D-dimer in Saudi Arabia population within the Emergency department and it clearly highlights their accuracy and diagnostic values. In this study, we aimed to investigate the causes of elevated D-dimer levels in patients presenting to a large emergency department in Saudi Arabia.

The results of this study revealed that majority of patients with D-dimer levels were labeled as “others” (78.3%). This group likely includes patients with D-dimer elevations due to non-thrombotic causes, such as infections, inflammation, or chronic diseases. These findings are consistent with previous studies demonstrating that D-dimer can be increased in various conditions, including infections and trauma [3, 13, 14]. Nevertheless, this study showed that D-dimer in respiratory infections had poor discriminatory value with AUC value of 0.6. Among the specific disease categories examined in this study, PE and DVT demonstrated acceptable discriminatory performance, with area under the curve (AUC) values of 0.844 and 0.795, respectively. The optimal cut-off points for PE and DVT were determined to be 1.17 g/L and 1.05 g/L, respectively. These findings align with a multicenter study done in the US emergency departments regarding D-dimer use in the diagnosis and exclusion of VTE in low-risk patients [15]. Interestingly, the finding in this study also assessed the discriminatory abilities of D-dimer levels for other diseases, such as aortic dissection, ACS, systemic lupus erythematosus (SLE) and stroke. Aortic dissection demonstrated acceptable discrimination (AUC = 0.737), suggesting a potential role for D-dimer in ruling out this life-threatening condition in low-risk patients which is in line with a meta-analysis conducted to assess D-dimer in acute aortic dissection [16]. However, it is worth noting that only 0.3% of our population were eventually diagnosed with aortic dissection. ACS showed poor discriminatory performance in our study (AUC = 0.52), indicating limited usefulness of D-dimer in this context, which contradicts previous study that assessed the diagnostic and prognostic value of D-dimer in ACS in which their findings proposed high D-dimer values of acceptable discrimination (AUC = 0.729) [6]. D-dimer was found to be of poor discriminatory value in our SLE patient which contraindicates the current literature that says D-dimer levels correlate with the disease severity [17]. Our results show that D-dimer levels in stroke patient has no discriminatory value (AUC = 0.614). Moreover, a study done in 2019 aimed to assess the usefulness of D-dimer in the work-up of stroke found that it may be of beneficial use in the stroke risk evaluation in cancer patients [18]. These results suggest that the utility of D-dimer in diagnosing or ruling out these specific diseases may be limited.

Our results highlight the variation in D-dimer levels across different diseases. Consistent with previous studies, we observed higher median D-dimer levels in patients with PE and DVT than most diseases [3]. Furthermore, our study revealed that D-dimer levels were significantly elevated in patients with aortic aneurysm and leukemia, with AUC values of 0.963 and 0.848, respectively. These findings are in line with previous research indicating the potential utility of D-dimer as a diagnostic marker in these conditions, however, it’s worth mentioning that in our study population only 3 patients were diagnosed to have aortic aneurysm [19, 20]. This study’s findings might be influenced by the ongoing COVID-19 pandemic, which has demonstrably impacted D-dimer ordering and interpretation in Saudi Arabia. Studies like Al-Qahtani et al. 2021 [21] suggest an increase in D-dimer tests due to COVID’s link to hypercoagulability, potentially influencing the number of measurements. Furthermore, Alqahtani et al. 2022 [22] highlight elevated D-dimer levels as a consequence of COVID infection itself, potentially skewing interpretations in this study. The overlapping clinical features between COVID-19 and other illnesses further complicate interpretations, as D-dimer levels might not definitively distinguish between them (Al-Qahtani et al., 2021). Therefore, acknowledging the potential impact of COVID-19 on both D-dimer ordering and interpretation is crucial for a nuanced understanding of the study’s findings.

It is important to acknowledge the limitations to this study. First, our findings are based on data collected from a single emergency department in Saudi Arabia, which may limit the generalizability of the results to other populations. Additionally, the retrospective nature of the study design may introduce selection bias and confounding factors. Future prospective studies involving larger and diverse patient populations are warranted to validate our findings and provide more robust evidence. Furthermore, While this study provides valuable insights into D-dimer levels, it is crucial to acknowledge that 78.3% of the analyzed patients had other diagnosis or no clear diagnosis. This absence of information hinders our ability to confidently draw conclusions about the specific association between D-dimer levels and various clinical conditions across the entire population.

## Conclusion

This study provides valuable insights into the diverse causes of elevated D-dimer levels in emergency department patients. Notably, we found excellent discriminatory power of D-dimer for specific diagnoses, including PE, leukemia, and aortic aneurysm. However, it demonstrated poor or no discriminatory power for other conditions. These findings have potential implications for practices within our department and others by sharing D-dimer results and clinical context which can optimize the selection of appropriate further testing, facilitating a more efficient and patient-centered diagnostic journey.

## Data Availability

The data generated and analyzed to support the findings of this study was provided by King Abdulaziz Medical City under license and is not publicly available. Access to the data will be considered by Dr Mohammed Alshalhoub upon request with the permission of King Abdulaziz Medical City.
